# Associations between patterns of blood heavy metal exposure and health outcomes: insights from NHANES 2011–2016

**DOI:** 10.1186/s12889-024-17754-0

**Published:** 2024-02-22

**Authors:** Xiangyu Wang, Xinhao Han, Shufang Guo, Yujie Ma, Yafeng Zhang

**Affiliations:** 1 Institute for Hospital Management of Henan Province, The First Affiliated Hospital of Zhengzhou University, No.1 Jianshe Dong Road, ErQi District, Zhengzhou, 450000 China; 2 Party Committee Office, Chaoyang Hospital, Capital Medical University, Beijing, China; 3 Department of Biostatistics, School of Public Health, Harbin Medical University, Harbin, China; 4 Department of Hematology and Oncology, Children’s Hospital Affiliated to Zhengzhou University, Henan Children’s Hospital, Zhengzhou Children’s Hospital, Zhengzhou, China

**Keywords:** Blood heavy metal exposure, Health outcomes, Clustering analysis

## Abstract

**Background:**

Extensive research has explored the association between heavy metal exposure and various health outcomes, including malignant neoplasms, hypertension, diabetes, and heart diseases. This study aimed to investigate the relationship between patterns of exposure to a mixture of seven heavy metals and these health outcomes.

**Methods:**

Blood samples from 7,236 adults in the NHANES 2011–2016 studies were analyzed for levels of cadmium, manganese, lead, mercury, selenium, copper, and zinc. Cluster analysis and logistic regression identified three distinct patterns of mixed heavy metal exposure, and their associations with health outcomes were evaluated.

**Results:**

Pattern 1 exhibited higher odds ratios (ORs) for malignancy during NHANES 2011–2012 (OR = 1.33) and 2015–2016 (OR = 1.29) compared to pattern 2. Pattern 3 showed a lower OR for malignancy during NHANES 2013–2014 (OR = 0.62). For hypertension, pattern 1 displayed higher ORs than pattern 2 for NHANES 2011–2012 (OR = 1.26), 2013–2014 (OR = 1.31), and 2015–2016 (OR = 1.41). Pattern 3 had lower ORs for hypertension during NHANES 2013–2014 (OR = 0.72) and 2015–2016 (OR = 0.67). In terms of heart diseases, pattern 1 exhibited higher ORs than pattern 2 for NHANES 2011–2012 (OR = 1.34), 2013–2014 (OR = 1.76), and 2015–2016 (OR = 1.68). Pattern 3 had lower ORs for heart diseases during NHANES 2013–2014 (OR = 0.59) and 2015–2016 (OR = 0.52). However, no significant trend was observed for diabetes. All three patterns showed the strongest association with hypertension among the health outcomes studied.

**Conclusions:**

The identified patterns of seven-metal mixtures in NHANES 2011–2016 were robust. Pattern 1 exhibited higher correlations with hypertension, heart disease, and malignancy compared to pattern 2, suggesting an interaction between these metals. Particularly, the identified patterns could offer valuable insights into the management of hypertension in healthy populations.

**Supplementary Information:**

The online version contains supplementary material available at 10.1186/s12889-024-17754-0.

## Background

Environmental factors, including exposure to toxic chemicals, significantly impact the global burden of diseases. According to data from the World Health Organization (WHO), these factors contribute to over 25% of the total burden [1]. Prolonged, excessive, or substantial exposure to heavy metals is associated with systemic diseases [2]. While increased awareness and preventive measures have reduced cases of heavy metal poisoning, the potential for accumulation of these metals in the body remains. However, there is currently a lack of standardized methods to precisely delineate levels of exposure to heavy metals. This underscores the necessity for effective differentiation to enhance public health management and promotion.

Lead (Pb), cadmium (Cd), and mercury (Hg) are among the primary environmental pollutants due to their significant impact on environmental contamination and health [3]. Pb, a widely distributed heavy metal, can induce neurological, hematological, renal, cardiovascular, and reproductive damage when ingested, inhaled, or absorbed through the skin [4, 5]. Even low-level exposure to Pb has been linked to hypertension [6]. Cd is associated with various diseases, including hypertension, diabetes, cancers, and coronary artery disease [7, 8]. Hg primarily exerts adverse effects on multiple organ systems, including the nervous, endocrine, reproductive, and embryonic development systems [9]. Studies indicate that combined exposure to Pb, Cd, and Hg demonstrates synergistic toxic effects, surpassing the effects of individual metals or combinations of two metals [10]. Studies on the correlation between heavy metals and human health have notably focused on malignant neoplasms, heart disease, hypertension, and diabetes. These diseases represent significant global public health challenges facing humanity. They profoundly impact patients’ quality of life and overall health, while also imposing a substantial burden on healthcare systems and socioeconomic structures worldwide. Estimates indicate that the annual economic impact of hypertension in the United States ranges from $138 billion to $198 billion [11], while heart disease incurs an approximate annual economic impact of $219 billion [12]. Additionally, the estimated annual total cost of diabetes stands at around $327 billion [13]. According to forecasts, the projected economic cost of cancer in the United States between 2020 and 2050 is anticipated to reach $5.3 trillion [14].

Research indicates that Pb exposure can result in hypertension and cardiovascular diseases by promoting oxidative stress, stimulating the renin-angiotensin system, and downregulating nitric oxide, among other biological mechanisms [15]. Pb can also foster the development of diabetes through oxidative stress reactions and alterations in intracellular signaling pathways [16]. Recent studies suggest that Cd might mimic steroid hormones like androgens and estrogens, supporting its potential role in hormone-related cancer development [17]. Cd may also induce several atherosclerotic effects by increasing coronary artery calcification, endothelial lipoprotein retention and oxidation, endothelial dysfunction, and promoting thrombus formation while inhibiting fibrinolysis [18]. Furthermore, Cd can impair pancreatic tissue, leading to excessive stimulation of gluconeogenesis in target tissues (especially adipose tissue), reduced insulin secretion, and insulin resistance, consequently reducing glucose uptake [18]. Mercury’s overall impact on blood vessels includes increased oxidative stress and inflammation, decreased oxidative defense capability, thrombus formation, impaired vascular smooth muscle function, endothelial dysfunction, abnormal blood lipid profiles, as well as immune and mitochondrial dysfunction [19].

Nutrient metals such as copper (Cu), manganese (Mn), selenium (Se), and zinc (Zn) within heavy metals have gained considerable attention. The U.S. Food and Drug Administration (FDA) has set Daily Values (DVs) for essential nutrient metals. These values suggest a recommended daily intake of 0.9 mg of Cu, 2.3 mg of Mn, 55 mcg of Se, and 11 mg of Zn for adults and children aged 4 years and older [20]. Studies indicate that imbalances in Cu, Mn, Zn, and Se are associated with hypertension, heart disease, diabetes, and cancer [21–26]. Excessive Cu levels trigger oxidative stress, contributing as one of the causal factors in the development of type 2 diabetes, whereas Zn exhibits the ability to activate pivotal molecules involved in cellular signaling, thereby ensuring the stability of glucose levels [22]. Reduced Se levels negatively impact redox regulation, thyroid hormone metabolism, and calcium flux while increasing atherosclerosis and oxidative stress [27]. Research has also found a positive correlation between Mn and blood pressure, and a U-shaped relationship with diabetes [28, 29]. Low Mn levels can inhibit pancreatic insulin synthesis, enhance degradation, decrease glucose transport in fat cells [30]. Excessive Mn disrupts the antioxidant activity of MnSOD, promoting ROS production, leading to elevated oxidative stress and inflammation levels [30]. Furthermore, exposure to moderate levels of Mn and high levels of Se can counteract Pb-induced increases in blood pressure [31].

Previous research primarily focused on examining the impact of individual metal exposures on health, but there is growing interest in understanding the cumulative effects of simultaneous exposure to multiple metals on health. Toxic metals such as Pb, Cd and Hg can inflict multi-organ damage even at low-level exposure, whereas increased exposure to essential nutrient metals like Cu, Mn, Se, and Zn can promote health. Therefore, categorizing metals based on their patterns of exposure in the human body aids in delineating the synergistic effects of essential and toxic metals on health. This study aims to investigate the relationship between various patterns of exposure to multiple heavy metals and significant health outcomes in American adults, including hypertension, diabetes, malignant neoplasms, and heart disease.

## Material and methods

### Study population

The National Health and Nutrition Examination Survey (NHANES) is an ongoing research program designed to assess the health and nutrition status of a representative population of noninstitutionalized individuals in the United States. The NHANES survey follows a multistage, stratified sampling approach and has been conducted biennially since 1999. For our study, we utilized publicly available NHANES data from the years 2011–2012, 2013–2014, and 2015–2016. The study focused on individuals aged 20 years and above. To ensure data completeness, participants with missing information on heavy metals in blood (Cd, Mn, Pb, Hg, Se, Cu, and Zn), malignancies, and severe cardiovascular diseases (e.g., previous myocardial infarction, heart failure, or stroke) were excluded from the analysis. Ultimately, 7,236 participants (3,728 males and 3,508 females) were included in the final analysis. The NHANES study and its protocols were approved by the Institutional Review Board of the National Center for Health Statistics, Centers for Disease Control and Prevention. All participants provided informed consent before participating in the survey. For detailed information regarding the study methodology, it can be accessed on the NHANES website at www.cdc.gov/nchs/nhanes/irba98.html.

### Measurement of heavy metals in blood

Biospecimens for Pb, Cd, Mn, Hg, Se, Cu, and Zn in blood were collected at the mobile examination center (MEC). Trained phlebotomists at the MEC conducted venipuncture to collect non-fasting blood samples, ensuring a minimum volume of 0.25 ml per vial. These samples were subsequently processed, stored, and shipped to various laboratories across the United States for analysis. The heavy metal concentrations in whole blood specimens (Cd, Mn, Hg, Pb, Se, Cu, and Zn) were directly determined via mass spectrometry following a simple dilution preparation step. Rigorous scrutiny was applied to the measured values of heavy metals, with any incomplete or improbable data sent back to the laboratories for confirmation. The lowest detection limits were as follows: Cd (0.10 µg/L), Mn (0.99 µg/L), Hg (0.28 µg/L), Pb (0.07 µg/dL), Cu (2.5 µg/dL), Zn (2.9 µg/dL), and Se (4.5 µg/dL). Values falling below the detection limit were calculated using the square root of 2 divided by the lowest detection limit. Units of Cd, Mn, and Hg (µg/L) were converted to nmol/L using conversion factors of 8.897, 18.202, and 4.99, respectively. Pb concentrations in µg/dL were converted to µmol/L by multiplying by 0.0483, while Se concentrations in µg/L were converted to µmol/L using a factor of 0.0127. Cu concentrations in µg/dL were converted to µmol/L by multiplying by 0.1570, and Zn concentrations in µg/dL were converted to µmol/L by multiplying by 0.1530. Sample weights were not factored into the analysis due to the limited number of replicates in which heavy metals were measured. For detailed instructions on specimen collection and processing, publicly available information can be accessed on the NHANES website, ensuring transparency and reproducibility.

### Covariate definition

Demographic factors were collected through household interviews, encompassing sex (male and female), age (recorded as a continuous variable), ethnic origin (Mexican-American, Hispanic, non-Hispanic White, African American, and other races), and education level (categorized as high school and below [≤ high school] or some college and above [≥ college]). Smoking status was self-reported and categorized as never, current, or former smoker. Additionally, participants’ body mass index (BMI) was determined using height and weight measurements obtained during the medical examination. BMI values were then categorized as follows: normal [< 25.0 kg/m^2^], overweight [25.0–29.9 kg/m^2^], or obese [≥ 30.0 kg/m^2^].

### Outcome assessment

Systolic blood pressure (SBP) and diastolic blood pressure (DBP) were determined as the average of four measurements. Hypertension was diagnosed based on the use of anti-hypertensive treatment, or when the mean SBP was ≥ 140 mmHg or the mean DBP was ≥ 90 mmHg at baseline. Diabetes mellitus was confirmed either by the use of anti-hyperglycemic therapy or when the HbA1c level was ≥ 6.5%. A history of heart failure, coronary artery disease, stroke, angina pectoris, or myocardial infarction was self-reported, and the presence of any one of these conditions was considered indicative of heart diseases. Malignant neoplasms were identified when physicians were informed of a cancer diagnosis or any malignancy.

### Statistical analysis

The data were analyzed and presented based on NHANES year (NHANES 2011–2012, 2013–2014, and 2015–2016). Continuous variables were expressed as mean ± standard deviation (SD), while categorical variables were presented as percentages. Due to the skewed nature of metal concentrations in blood, their distribution was described using the median (interquartile range, IQR). To achieve a more normal distribution for analysis purposes, a logarithmic transformation of the metal concentrations was conducted.

Clustering analysis was employed to identify patterns of exposure to different heavy metals. This unsupervised learning technique is used to discern natural groupings of observations based on the inherent structure of the dataset, thereby reducing highly multivariate datasets. Cluster analysis categorizes individuals into relatively uniform groups, enabling direct comparison among these groups. For the identification of blood heavy metal exposure patterns, the FASTCLUS program was utilized. Participants were classified based on similarities in their blood heavy metal exposure using the K-means method, which is one of the most commonly used clustering algorithms. This method computes cluster centers based on least squares estimation. Prior to the analysis, the number of clusters needed to be specified. Initially, the FASTCLUS program generated 20 clusters, then temporarily removed participants from clusters containing fewer than 5 individuals. From this subset, the number of clusters varied from 2 to 6 to ascertain the optimal number that would provide a reasonably sized and interpretable solution. Ultimately, three clustering solutions were selected to represent the blood heavy metal exposure patterns observed within this population.

The sample matrix eigenvectors were calculated, and the top three eigenvariables extracted 91.10%, 91.48%, and 91.47% of the sample information in 2011–2012, 2013–2014, and 2015–2016, respectively. These eigenvariables served to identify three patterns of metal concentrations in blood using a stepwise clustering method. Differences among the three patterns across NHANES years were compared using a nonparametric test for multiple group comparisons (Kruskal-Wallis test). Additionally, logistic regression analysis was employed to investigate the relationship between these patterns and outcome variables (such as malignant neoplasms, hypertension, heart diseases, and diabetes), while accounting for covariates. The evolutionary characteristics of the three patterns concerning the outcome variables were visualized using a Sankey plot. Statistical analyses were conducted using SAS (version 9.4) and R software (version 3.3.2) (http://www.r-project.org/), with statistical significance set at *P* < 0.05.

## Results

### Baseline characteristics across various NHANES years

Table 1 illustrates the baseline characteristics of NHANES samples collected during 2011–2012, 2013–2014, 2015–2016, and the combined years. It presents median values (IQR) and percentages for demographic factors such as age, sex, BMI, race, education, smoking status, and the prevalence of malignant neoplasms, hypertension, heart diseases, and diabetes. No statistically significant differences were observed when comparing these characteristics across the examined years. Table 2 showcases median values (IQR) of metal concentrations (Cd, Mn, Pb, Hg, Se, Cu, Zn). None of the inter-year comparisons yielded statistical significance. These findings suggest consistently obtained samples, ensuring a balanced representation across different periods.

**Table 1:** Participant characteristics in NHANES 2011–2012, 2013–2014, and 2015–2016

Characteristic	2011–2012 *N* = 2314	2013–2014 *N* = 2505	2015–2016 *N* = 2417	Overall *N* = 7236
Age (IQR)	46 (35–59)	46 (36–59)	47 (36–60)	46 (36–59)
Sex (%)				
Male	1191 (51.5)	1289 (51.5)	1248 (51.6)	3728 (51.5)
Female	1123 (48.5)	1216 (48.5)	1169 (48.4)	3508 (48.5)
BMI (kg/m^2^, %)				
≤ 25	683 (38.7)	677 (35.7)	629 (34.2)	1989 (36.2)
25–25.9	567 (32.1)	614 (32.4)	610 (33.2)	1791 (32.6)
≥ 30	514 (29.1)	606 (31.9)	600 (32.6)	1720 (31.3)
Race (%)				
Mexican	283 (12.2)	426 (17.0)	460 (19.0)	1169 (16.2)
Hispanic	239 (10.3)	235 (9.38)	333 (13.8)	807 (11.1)
White	775 (33.5)	972 (38.8)	754 (31.2)	2501 (34.6)
Black	616 (26.6)	512 (20.4)	491 (20.3)	1619 (22.4)
Other	401 (17.3)	360 (14.4)	379 (15.7)	1140 (15.7)
Education (%)				
High school or below	703 (43.0)	764 (43.6)	790 (45.7)	2257 (44.2)
College graduate or above	931 (57.0)	987 (56.4)	937 (54.3)	2855 (55.8)
Smoking status (%)				
Current	253 (36.7)	289 (37.3)	246 (32.8)	788 (35.6)
Former	58 (8.42)	75 (9.69)	87 (11.6)	220 (9.9)
Never	378 (54.9)	410 (53.0)	417 (55.6)	1205 (54.5)
Diabetes (%)	213 (9.20)	207 (8.26)	269 (11.1)	689 (9.5)
Hypertension (%)	589 (32.5)	652 (33.4)	614 (32.3)	1855 (32.7)
Heart diseases (%)	148 (6.40)	163 (6.51)	174 (7.20)	485 (6.7)
Malignant neoplasms (%)	132 (5.70)	160 (6.39)	161 (6.66)	453 (6.3)

*Abbreviations*: *N* Number of cases, *IQR* Interquartile range, *BMI* Body mass index

**Table 2:** Metal concentrations in NHANES 2011–2012, 2013–2014, and 2015–2016

Metal	2011–2012 Median (IQR)	2013–2014 Median (IQR)	2015–2016 Median (IQR)	Overall Median (IQR)
Cd	0.26 (0.11–0.48)	0.22 (0.12–0.43)	0.23 (0.13–0.42)	0.23 (0.12–0.44)
Hg	0.65 (0.33–1.46)	0.62 (0.33–1.33)	0.60 (0.34–1.22)	0.62 (0.33–1.34)
Pb	0.91 (0.58–1.52)	0.81 (0.51–1.32)	0.78 (0.50–1.36)	0.83 (0.53–1.40)
Cu	112.60 (97.90–132.60)	115.10 (99.60–133.40)	114.70 (99.00–133.20)	114.30 (98.80–133.00)
Mn	9.33 (7.52–11.84)	9.48 (7.69–11.94)	9.80 (7.92–12.35)	9.56 (7.71–12.04)
Se	124.30 (113.90–136.30)	126.80 (116.10–138.80)	125.60 (115.70–135.50)	125.60 (115.30–136.80)
Zn	81.05 (72.40–91.10)	80.30 (71.30–90.20)	80.50 (70.40–90.30)	80.60 (71.40–90.50)

*Abbreviation*: *IQR* Interquartile range

### Identification of patterns through cluster analysis

**Fig. 1:**
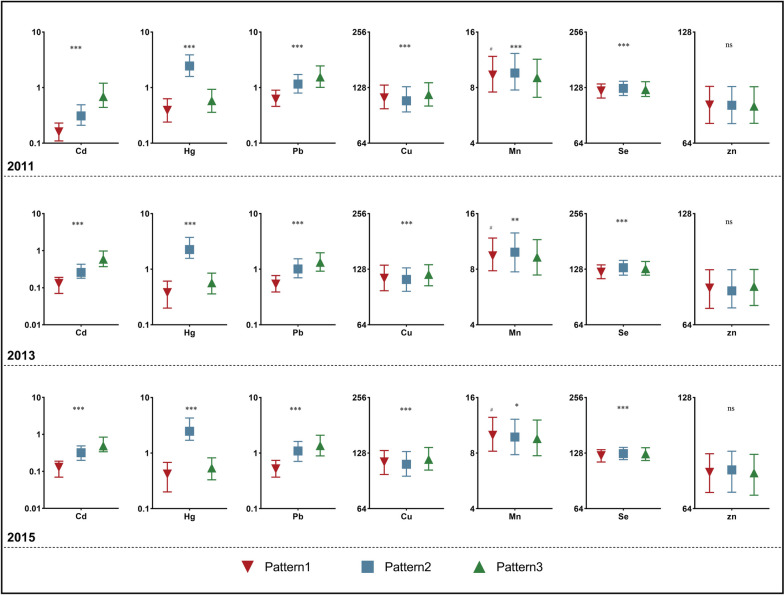
Patterns of heavy metal concentrations in NHANES 2011–2012, 2013–2014, and 2015–2016

The cluster analysis results revealed a three-cluster solution effectively distinguishing heavy metal concentrations in blood. Pattern 1, labeled ‘low Cd-Pb-Hg-Se’, consistently exhibited low exposure levels to Cd, Pb, Hg, and Se. Pattern 2, termed ‘low Cu & high Hg-Se’, showed low Cu exposure but high Hg and Se exposure. Pattern 3 indicated high exposure to Cu, Cd, and Pb, referred to as ‘high Cu-Cd-Pb’. These patterns remained consistent throughout NHANES 2011–2015, demonstrating stability. Figure 1 visually demonstrates the variations in heavy metal concentrations among these patterns. As depicted in Fig. 1 and detailed in Tables S1-S3, all heavy metal concentrations, except Zn, exhibited statistically significant differences among the patterns. Notably, in NHANES 2011–2012 and 2013–2014, Mn levels were elevated in pattern 2 and reduced in pattern 3. However, in NHANES 2015–2016, Mn exposure increased in pattern 1 while remaining lower in pattern 3.

### Correlation between three patterns and outcome variables

**Fig. 2:**
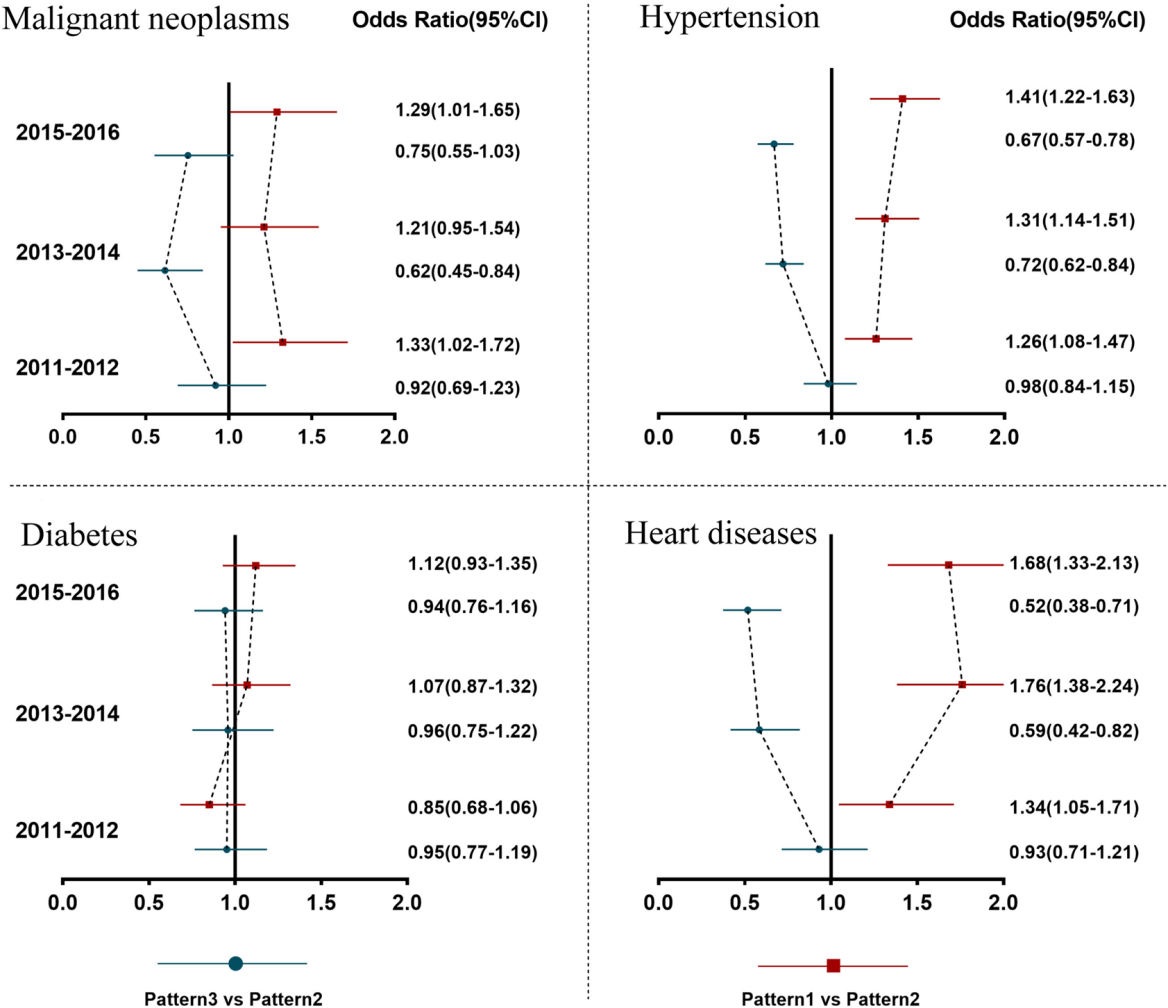
Association of 3 patterns with malignant neoplasms, hypertension, diabetes and heart diseases adjusted for age (continuous, years), gender (male or female), ethnicity (non-Hispanic white, non-Hispanic black, Hispanic, or other), with additional adjustment for BMI (continuous, kg/m^2^) in all models

Figure 2 depict the relationship between the identified patterns and adverse outcome events. In the case of malignant neoplasms, participants in pattern 1 exhibited odds ratios (ORs) of 1.29 (95% CI 1.01–1.65) for 2015–2016 and 1.33 (95% CI 1.02–1.72) for 2011–2012 when compared to pattern 2. Pattern 3 showed a significant association in 2013–2014, with an OR of 0.62 (95% CI 0.45–0.84) versus pattern 2. Regarding hypertension, participants in pattern 1 displayed ORs of 1.41 (95% CI 1.22–1.63), 1.31 (95% CI 1.14–1.51), and 1.26 (95% CI 1.08–1.47) for the years 2015–2016, 2013–2014, and 2011–2012, respectively, compared to pattern 2. Conversely, participants in pattern 3 exhibited ORs of 0.67 (95% CI 0.57–0.78) and 0.72 (95% CI 0.62–0.84) for the years 2015–2016, and 2013–2014. No statistically significant trends were observed for the ORs concerning pattern 1 and pattern 3 compared to pattern 2 for diabetes. Regarding heart diseases, participants in pattern 1, contrasted with those in pattern 2, had ORs of 1.68 (95% CI 1.33–2.13), 1.76 (95% CI 1.38–2.24), and 1.34 (95% CI 1.05–1.71) for 2015–2016, 2013–2014, and 2011–2012, respectively. Pattern 3 exhibited significance solely in the years 2015–2016 and 2013–2014, with ORs of 0.52 (95% CI 0.38–0.71) and 0.59 (95% CI 0.42–0.82), respectively.

**Fig. 3:**
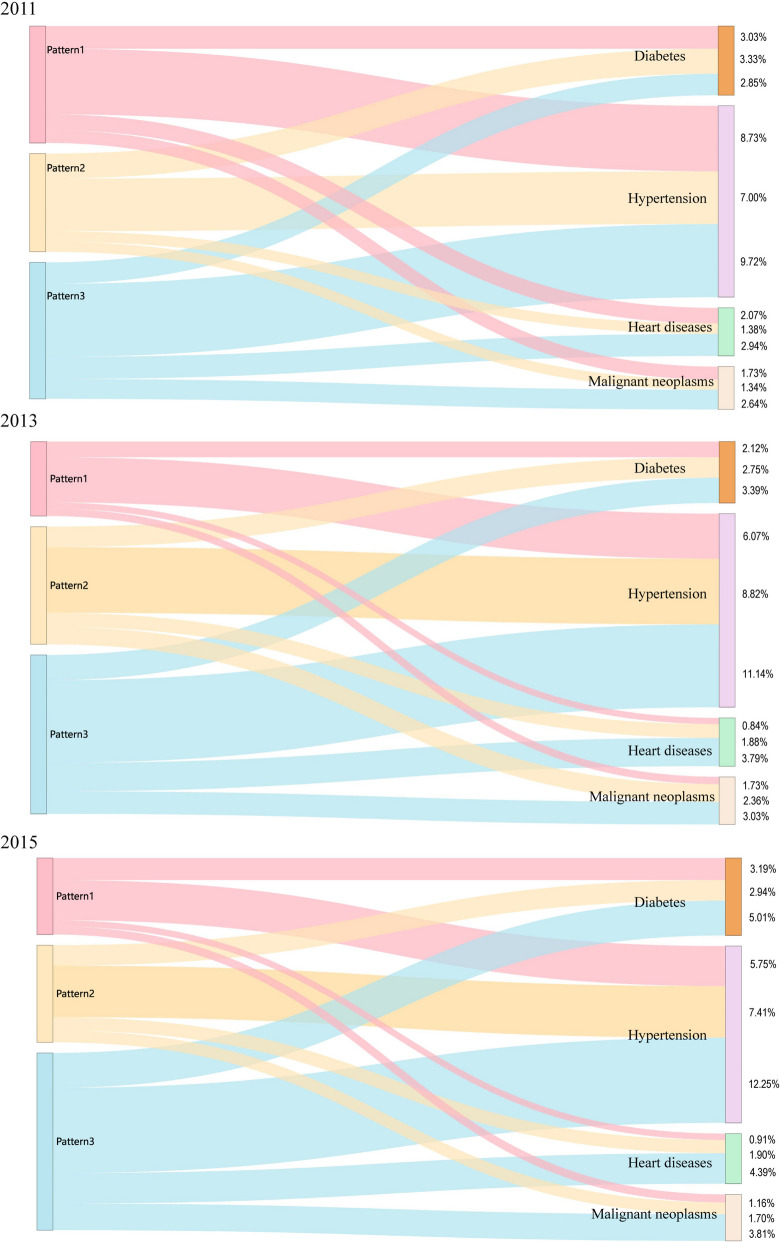
Sankey diagram illustrating the relationship between three patterns and malignant neoplasms, hypertension, diabetes and heart disease

Figure 3 illustrates that Pattern 1 comprises 37.33% of the participants. Within this group, 3.82%, 4.62%, 8.34%, and 20.55% were associated with heart diseases, malignant neoplasms, diabetes, and hypertension, respectively. Pattern 2 includes 42.81% of the subjects, with 5.16%, 5.40%, 9.02%, and 23.23% linked to heart diseases, malignant neoplasms, diabetes, and hypertension, respectively. Pattern 3 accounted for 64.96% of the subjects, among whom 11.12%, 9.48%, 11.25%, and 33.11% were associated with heart diseases, malignancy, diabetes mellitus, and hypertension, respectively. These results suggest an association with hypertension among the major participants in the three patterns.

## Discussion

In this study, we identified three distinctive patterns of heavy metal concentrations in the human body. These patterns diverged from prior research by encompassing both essential and toxic metals, labeled as “low Cd-Pb-Hg-Se”, “low Cu & high Hg-Se”, and “high Cu-Cd-Pb”. When compared to pattern 2, pattern 3 exhibited an inverse association with malignancy in NHANES 2013–2014, while pattern 1 showed an elevated likelihood of malignancy in NHANES 2011–2012 and 2015–2016. Furthermore, pattern 1 displayed an increased prevalence of hypertension and heart diseases over time, whereas pattern 3 demonstrated a decreased prevalence of these conditions. Neither pattern 1 nor pattern 3 showed a statistically significant association with the likelihood of diabetes compared to pattern 2. Hypertension was closely linked to all three patterns concerning adverse outcomes. This study contributes to our understanding of the impact of different metal patterns on health outcomes and the underlying mechanisms.

Previous epidemiological studies have often focused on either the cumulative impacts of toxic metals or the individual effects of essential nutritional metals, neglecting the intricate interplay between exposures to both types [32]. In our study, we discovered that multiple metals in the human body interact with one another, giving rise to three distinct patterns that establish a stable and balanced state. For instance, we observed that pattern 1 characterized by lower levels of exposure to Pb, Cd, Hg, and Se. These heavy metals Pb, Cd, and Hg are widely acknowledged for their toxicity, even at minimal exposure levels, exerting significant impacts on the structure and function of various organs [33]. Conversely, Se is recognized as nature’s antidote to heavy metal toxicity and an essential trace element [34]. Their combined presence may potentially produce a synergistic effect that shields the body from certain types of harm.

Additionally, we found that low exposure to Cu coincided with high exposure to Hg and Se, forming pattern 2. Previous studies showed that there is a molecular interaction between Hg and Se, and that the two form stable coordination compounds, and that supplementation with Se ameliorates the toxicity of Hg compounds [35, 36]. Cu, being an essential nutritional metal, serves as a cofactor for numerous redox enzymes and maintains bodily homeostasis [37]. Our findings suggest that diminished exposure to Cu may foster antagonistic interactions between Hg and Se, thereby establishing a novel equilibrium.

Nonetheless, it is imperative to acknowledge that high doses of Cu can be toxic, leading to adverse effects such as nausea, vomiting, stomach cramps, and diarrhea [38, 39]. Similarly, elevated levels of Cd and Pb can have severe adverse health impacts on various organs and tissues [40]. Although uncertainties remain regarding the reference values of Cu within the nutritional range [41], our results indicate that high exposure to Cu, Cd, and Pb may induce additive threatening effects. These three identified patterns offer novel insights into unraveling the intricate interactions between different metal components.

The study also explored the relationship between the three patterns and the incidence rates of hypertension, heart diseases, and malignancy. Pattern 1 was associated with higher incidences of hypertension, heart diseases, and malignancy, while Pattern 3 was linked to lower incidences of hypertension and heart diseases. Previous study indicated an association between exposure to Cu, Cd, Pb and increased incidence rates of cardiovascular diseases and coronary artery diseases [42]. Cu, believed to promote atherosclerosis by facilitating the oxidation of low-density lipoprotein cholesterol, has been associated with cardiovascular diseases [42]. Recent studies have shown a connection between exposure to heavy metals and malignancy, suggesting a higher incidence of cancer with increased levels of heavy metals [43, 44]. This study confirms that exposure to Cu, Cd, Pb may also elevate the incidence rates of hypertension and malignancy.

However, the association between these three patterns and diabetes was not observed in our study. While some research indicates that variations in toxic and trace elements may be linked to the occurrence and onset of diabetes, the findings across studies are inconsistent [45, 46]. Specifically, high Cd exposure is associated with an increased risk of type 2 diabetes, and elevated levels of total Cu and ceruloplasmin have been found in patients with type 1 diabetes [47, 48]. Nevertheless, the evidence linking Hg exposure to the development of diabetes is limited and uncertain [49]. The use of Pattern 2 as the reference level (high Hg and Se and low Cu exposure) in the study might also be a potential reason for the nonsignificant association between Patterns 1 and 3 and diabetes.

Furthermore, we found that a substantial proportion of subjects distinguished by the 3 patterns flowed toward hypertension in the health outcome. This inclination was most pronounced for pattern 1 and exhibited increased stability over years. These results imply that the impact of metal combinations on hypertension might not solely result from additive effects or dominance by a single metal element but could involve intricate interactions among metal mixtures.

There are several limitations that should be considered in this study. Firstly, the associations observed between metal mixture patterns and health outcomes relied on cross-sectional data from NHANES, which introduces the potential for reverse causality. It is plausible that adverse health outcomes might influence the excretion of heavy metals in the body, potentially leading to confounding effects. Secondly, our classification of metal concentrations focused on seven specific heavy metals. Considering the high correlation and potential interactions among different metals, the inclusion of additional heavy metals would improve our understanding of the relationship between exposure to multiple metal mixtures and health outcomes. Thirdly, the identified patterns of metal concentrations represent recent exposures, as heavy metals possess varying half-lives in the body. Further investigation is necessary to explore the dose-response relationships between cumulative metal concentrations and health outcomes. Lastly, elucidating the biological mechanisms underpinning the effects of metal mixture patterns on adverse health outcomes requires additional research and clarification.

## Conclusion

In conclusion, the study utilized cluster analysis to classify seven heavy metals found in the human body, encompassing both toxic elements (Pb, Hg, Cd) and essential nutrients (Cu, Mn, Se, Zn), into three distinct patterns. Notably, pattern 1, characterized by a mixture of these metals, exhibited associations with hypertension, heart diseases, and malignancy. Additionally, our findings suggested that the interaction between these metals had a more pronounced impact on hypertension compared to other adverse health outcomes. It is imperative to conduct prospective studies to validate these findings, as they hold significant implications for public health. Confirming these results could inform population health promotion strategies and aid in the formulation of effective environmental guidelines.

### Supplementary Information

**Additional file 1: Table S1.** Analysis of Differences in Heavy Metal Concentrations Among Three Patterns in NHANES 2011-2012. **Table S2.** Analysis of Differences in Heavy Metal Concentrations Among Three Patterns in NHANES 2013-2014. **Table S3.** Analysis of Differences in Heavy Metal Concentrations Among Three Patterns in NHANES 2015-2016.

**Additional file 2:** Patterns 1 and 3 as References for Analyzing Relationships Between Other Patterns and Health Outcomes.

## Data Availability

The datasets generated and/or analyzed during the current study are available in the [National Health and Nutrition Examination Survey] repository, [https://wwwn.cdc.gov/Nchs/Nhanes/continuousnhanes/default.aspx].
