# Supplementary material for: Associations between patterns of blood heavy metal exposure and health outcomes: insights from NHANES 2011–2016
Source: BMC Public Health. 2024 Feb 22;24:558. doi: 10.1186/s12889-024-17754-0 (PMC10882930; doi:10.1186/s12889-024-17754-0)
Supplement: Supplementary file 1 — Additional file 1: Table S1. Analysis of Differences in Heavy Metal Concentrations Among Three Patterns in NHANES 2011-2012. Table S2. Analysis of Differences in Heavy Metal Concentrations Among Three Patterns in NHANES 2013-2014. Table S3. Analysis of Differences in Heavy Metal Concentrations Among Three Patterns in NHANES 2015-2016. [file 12889_2024_17754_MOESM1_ESM.docx]

**Supplementary material**

**Table S1 Analysis of Differences in Heavy Metal Concentrations Among Three Patterns in NHANES 2011-2012**

| Variable | Pattern1 | Pattern2 | Pattern3 | *P* Value |
| --- | --- | --- | --- | --- |
| Cd | 0.16(0.11-0.23) | 0.31(0.21-0.49) | 0.69(0.44-1.20) | <.0001 |
| Hg | 0.39(0.24-0.63) | 2.45(1.59-3.89) | 0.58(0.36-0.93) | <.0001 |
| Pb | 0.62(0.46-0.90) | 1.16(0.80-1.72) | 1.55(1.01-2.46) | <.0001 |
| Cu | 112.60(98.30-132.20) | 108.60(94.50-129.40) | 117.35(101.75-136.05) | <.0001 |
| Mn | 9.36(7.57-11.82) | 9.60(7.77-12.25) | 9.06(7.09-11.40) | 0.0008 |
| Se | 122.90(112.40-134.00) | 126.70(116.00-138.50) | 124.90(114.65-137.75) | <.0001 |
| Zn | 81.20(72.40-91.20) | 81.10(72.30-91.10) | 80.60(72.45-91.00) | 0.8779 |

**Table S2 Analysis of Differences in Heavy Metal Concentrations Among Three Patterns in NHANES 2013-2014**

| Variable | Pattern1 | Pattern2 | Pattern3 | *P* Value |
| --- | --- | --- | --- | --- |
| Cd | 0.13(0.07-0.19) | 0.26(0.18-0.43) | 0.59(0.37-0.98) | <.0001 |
| Hg | 0.38(0.20-0.61) | 2.26(1.57-3.74) | 0.57(0.36-0.85) | <.0001 |
| Pb | 0.54(0.39-0.77) | 1.01(0.70-1.54) | 1.33(0.92-1.98) | <.0001 |
| Cu | 114.40(97.90-134.60) | 112.70(97.20-130.10) | 119.85(104.10-135.40) | <.0001 |
| Mn | 9.45(7.85-11.80) | 9.89(7.74-12.58) | 9.30(7.44-11.57) | 0.0022 |
| Se | 123.40(113.70-135.10) | 130.40(118.80-142.90) | 129.00(118.80-140.90) | <.0001 |
| Zn | 80.45(70.90-90.20) | 79.10(71.10-90.20) | 81.30(72.20-90.40) | 0.1 |

**Table S3 Analysis of Differences in Heavy Metal Concentrations Among Three Patterns in NHANES 2015-2016**

| Variable | Pattern1 | Pattern2 | Pattern3 | *P* Value |
| --- | --- | --- | --- | --- |
| Cd | 0.13(0.07-0.19) | 0.32(0.20-0.49) | 0.49(0.34-0.85) | <.0001 |
| Hg | 0.42(0.20-0.68) | 2.47(1.70-4.28) | 0.54(0.33-0.82) | <.0001 |
| Pb | 0.52(0.37-0.74) | 1.09(0.71-1.62) | 1.36(0.89-2.09) | <.0001 |
| Cu | 114.60(98.00-132.20) | 111.40(95.90-130.50) | 118.40(103.50-137.20) | <.0001 |
| Mn | 9.99(8.19-12.50) | 9.78(7.84-12.21) | 9.59(7.74-12.08) | 0.0291 |
| Se | 123.80(114.50-133.50) | 127.20(118.00-137.40) | 126.70(116.70-136.80) | <.0001 |
| Zn | 80.30(70.80-90.10) | 81.50(70.90-91.60) | 80.00(69.60-89.80) | 0.0692 |
